# Varieties of suffering in the clinical setting: re-envisioning mental health beyond the medical model

**DOI:** 10.3389/fpsyg.2023.1155845

**Published:** 2023-05-19

**Authors:** Paul T. P. Wong, Don Laird

**Affiliations:** ^1^Department of Psychology, Trent University, Peterborough, ON, Canada; ^2^Department of Psychology, Counseling & Criminology, Carlow University, Pittsburgh, PA, United States

**Keywords:** suffering, deep life, existential positive psychology, complete wellbeing, sustainable wellbeing

## Abstract

In this paper, we argue for the need to rethink mental health beyond the medical model because much of human suffering cannot be diagnosed by the DSM-5. During the pandemic and post-pandemic, people have learned to accept the fact that no one is immune from suffering. Given the universality and complexity of human suffering, it is natural for people to wrestle with existential questions such as “Why struggle when all life end in death?” and “How can one flourish when life is so hard?” Existential positive psychology (EPP or PP2.0) was developed to address these existential concerns. After explaining the inherent limitations of the medical model and the need for EPP as an alternative vision for mental health, we provide illustrative clinical cases to demonstrate the advantages of this broader existential framework for both case conceptualization and interventions. According to EPP, mental illness is reconceptualized as both deficiency in knowledge and skills in coping with the demands of life and deficiency in meeting the basic needs for livelihood and mental health, the Soul’s yearnings for faith, hope, and love. Finally, we introduce integrative meaning therapy as a therapeutic framework which can equip people with the needed skills to achieve healing, wholeness, and total wellbeing.

## Introduction

The era of coronavirus disease 2019 (COVID-19) has ushered in a global mental health crisis (De Kock et al., 2022; de la Rosa et al., 2022; Wong et al., 2022a). We need to rethink mental health beyond the medical model and the Diagnostic and Statistical Manual of Mental Disorders (DSM-5; American Psychiatric Association, 2013) because most human suffering cannot be diagnosed by the DSM-5. For example, many people suffer from the frequent mass killings (Metzl et al., 2021) and overloading of bad news (Huff, 2022), but this type of social suffering is not covered by the DSM-5.

After the pandemic, people have generally learned to accept the fact that no human being is immune from suffering. This creates an opening for a broader conversation regarding the various effects of suffering on mental health. Most people do not realize that suffering is not always bad for us, because suffering and wellbeing are intertwined in a complex way (Anderson, 2014). That is why a science of suffering is needed to develop a taxonomy of suffering and the different outcomes of suffering depending on how individuals react to it.

A preliminary taxonomy of suffering encompasses at least four kinds of suffering: physical suffering (physical injury or pain in one’s body), psychological suffering (the ego, painful emotions and inherent human limitations), social and interpersonal suffering (injustice, interpersonal conflicts and crimes; Kleinman et al., 1997; Moghaddam, 2022), and existential suffering (struggling with the ultimate concerns and unmet spiritual needs for meaning; Bates, 2016; Wong and Yu, 2021).

Given the universality and complexity of suffering, it is only natural for people to wonder “How could we find happiness when suffering is an inescapable part of life?” and “What is the point of striving when all life ends in death?” Neither existential philosophy nor positive psychology can by itself provide a complete answer to the human quest for meaning and happiness. We need a broader, integrative, and interdisciplinary framework to address the existential angst of common people regarding the ultimate concerns (Yalom, 1980), the meaning of life and suffering (Frankl, 1985), and the meaning of love in a multicultural society (Wong and Mayer, 2023). We also need an inviolable narrative of human beings’ role in the world and beyond (Feder, 2020). Existential positive psychology (EPP or PP2.0) was developed to provide a such holistic and interdisciplinary framework for mental health beyond medical model (Wong, 2020a).

In this paper we first explain the inherent limitations of the medical model in today’s complex and fragmented society and why EPP is both necessary and beneficial for both mental health. We then provide illustrative case studies to demonstrate the advantages of this broader alternative framework for case conceptualization and interventions. According to EPP, mental illness can be reconceptualized as both deficiency in knowledge and skills in coping with the demands and suffering of life and deficiency in meeting the basic needs for livelihood and mental health. Finally, we introduce integrative meaning therapy as a therapeutic framework to achieve healing, wholeness, and sustainable flourishing even in times of suffering.

### The medical model and the Diagnostic and Statistical Manual of Mental Disorders

The medical model is essentially a biomedical model which “posits that mental disorders are brain diseases and emphasizes pharmacological treatment to target presumed biological abnormalities” (Deacon, 2013). Accordingly by design, the Diagnostic and Statistical Manual of Mental Disorders (DSM) inherently implies that something is wrong with a person and that they are ill.

The stigmatizing effects of the DSM have been documented for a long time (Piner and Kahle, 1984; Corrigan and Penn, 1999). Diagnostic labels derived from the DSM can also lead clinicians to “implicitly adopt a disease model which may have negative consequences for the process of psychotherapy, such as less empathy for the client as a fellow human being.” (Honos-Webb and Leitner, 2001, p. 38) Recently, Raskin et al. (2022) found that nearly 90% of psychologists used the DSM despite being dissatisfied with it. For many people, their psychological problems can be attributed to the circumstances of their lives. Focusing only on the individual misses the complex relational context and malfunctioning social structures (Robbins et al., 2017). Therefore, the first goal of any therapist should be to recognize the source, nature and context of the client’s presenting problems.

Unfortunately, the DSM cannot assess the complex human experiences that are central to the human condition, such as existential loneliness or existential anxiety. Pathologizing these fundamental human experiences actually prevent the psychotherapist from offering the needed help. The cartesian approach is insufficient for realizing the complexities and nuances of the lived human experiences (Bradford, 2009).

Any manual bold enough to monopolize an entire profession inevitably lacks the necessary scope to include all the causes of psychological suffering, as we have alluded to in the introduction. It would be more helpful for the psychotherapist to understand clients’ struggles for reasons of living in an absurd world or their need for guidance and wisdom to resolve common problems such as how to relate better with their spouses and their bosses.

Likewise, cognitive-behavioral therapy (CBT) handbooks are promoted to explain away any kind of psychic pain in terms of irrational thinking, thus, either blaming or clinically gaslighting clients. In a post-pandemic world, this approach often ignores clients’ existential struggles for meaning and happiness and the macro problems such as climate change, internet scams, abuse of AI for personal gains, and the potential for international wars in Europe and Asia.

Mental health is more than an individual issue. It is also interpersonal, societal, cultural, and transcendental. Furthermore, it is not helpful to pathologize normal human reactions to complex and difficult life situations. Therefore, a new narrative of mental illness is needed to reduce the stigma by recognizing that other factors, such nature, society, and fate, are often beyond individual control and can negatively impact one’s mental health.

### What is EPP? Why is it a necessary and beneficial framework for mental health?

EPP is based on Wong’s five decades of research on suffering, clinical practice on suffering, and the integration of East and West such as the ancient Chinese wisdom of the dialectical principles of Yin and Yang (Wong and Cowden, 2022; Wong, n.d.a).

Culture plays an important role in shaping our attitude toward suffering. Traditionally, children in China were taught by their parents and schools that the most important lesson in life is the ability of “eat bitterness” [不吃苦, 不成人]. This simple Chinese proverb means that if you do not learn how to endure hardships, you will never amount to anything in life. An analogy is that if you do not develop deep roots, you cannot grow into a tall tree. To me (Wong), this is a truism, but this worldview is not widely accepted in the West.

In the Western culture, the dominant worldview is to enjoy life; if we focus on the positives, the negatives simply go away. In some way, it is desirable to be happy-go-lucky people, who have both the temperament and economic resources to enjoy life without much worry. The downside of this approach is that they are ill prepared when their comfortable life is disrupted by the inescapable storms of life, such as the pandemic, death of a loved one, bitter divorce, or terminal cancer.

Human nature does not change. At the deepest level, what is personal is also universal. The existential universals are the same for all cultures. These existential givens are our ultimate concerns, such as personal mortality, existential loneliness, the meaning of human existence, and the meaning of suffering. Repressed existential anxiety may manifest itself in other forms (Yalom, 1980).

Another existential universal is that we all have experienced the civil war between good and evil (Challa, 2020). Yet, it is painful to confront our dark side; as a result, we do not know how to relate to our Shadow, which is part of our true self (Perry, 2015). The lack of deep self-knowledge often leads to bad decisions. Denial or covering up our mistakes only make things worse.

Perhaps, the biggest existential challenge for anyone is going through the pain, isolation, and fears of the unknown during the last stage of life. As an 86-year-old man, I (Wong) have gone through near death experience more than once. More than 10 years ago, I was rushed to the hospital by an ambulance after collapsing in a poor of blood. I gave a blow-by-blow account of the horrors of being “to hell and back” (Wong, 2008). This experience led my discovery of mature happiness (Wong and Bowers, 2018).

Recently, I had another close encounter with death and went through all the painful procedures and aftermaths of surgery (Wong, 2023a). Strong belief in self-efficacy and all my research on successful aging was not enough to cope with the existential crisis of the end-of-life stage. One needs all the existential competences and all the spiritual and social support in order to go through the crisis with inner peace or equanimity (Wong and Yu, 2021; Wong, 2023b).

In view of the above, we need to recognize that suffering remains a missing link in wellbeing research (Anderson, 2014; Fowers et al., 2017; Soper, 2020; Clifton, 2022; Wong et al., 2022) and a promising direction of future research on human flourishing. According to EPP, the new science of flourishing through suffering involves not only research on different types of suffering, but also the processes and the outcomes of sustainable wellbeing.

The following represents some of the advantages of the EPP framework of integrating East and West and intertwining suffering and happiness, which expands wellbeing research beyond the binary approach:

1. The process of navigating the dialectical interactions between Yin-Yang in order to discover the adaptive balance or the middle way between positives and negatives (Wong, 2012a; Wong, 2016a; Wong and Cowden, 2022).
2. The process of transcending suffering, inherent limitations, and duality through self-transcendence (Kaufman, 2020; Wong, 2020a; Wong et al., 2021a,b).
3. The outcome of true positivity of seeing the light in the darkness, such as tragic optimism (Leung et al., 2021), existential gratitude (Jans-Beken and Wong, 2019), chaironic happiness (Wong, 2011), and mature happiness (Carreno et al., 2023). This type of happiness is characterized by achieving some kind of inner peace, balance and harmony through the difficult process of adapting to suffering or difficulty (Lomas, 2021).

There are hopeful signs of a paradigm shift (Harvard Human Flourishing Program, 2022; Wong et al., 2022a). Various recent publications (Bloom, 2021; Cain, 2022; Ho et al., 2022; Rashid and Brooks, 2022) also emphasize the need to integrate suffering for happiness and flourishing. Buddhist psychology has been the strongest advocate of ending suffering as the precondition for happiness. Its first noble truth is that life is suffering because our desires for carnal happiness and our ignorance of the impermanence of life (Thera, 2004; Targ and Hurtak, 2006; Cowden et al., 2021).

The need to embrace suffering and transform it into something meaningful is a recurrent theme in philosophy, literature, and religion (Heller, 2015). EPP is simply an extension of existential psychology (May, 1969; Yalom, 1980; Frankl, 1985) into a new science of suffering by developing a comprehensive account of the effects of suffering and its complex interactions with wellbeing (Wong, 2019; Wong et al., 2021b, 2022b). The following examples serve to illustrate the advantages of the broader EPP framework for mental health and psychotherapy.

## Illustrative examples of suffering in clinical settings^1^

Many of my (Wong) clients came to me because they were attracted to my meaning-centered therapy and counseling.

### Case one

Jackie suffered from depression. She was a 39-year-old attractive woman with a five-year-old son. Her husband was a very successful developer who worked 7 days a week and seldom came home for dinner. She used to work as a real state agent together with her husband; they used to struggle together in the early years of their marriage. Those were her happiest time of her life. After the birth of their first son, she became a stay-at-home housewife. Even though she was able to hire two helpers and had lots of time to do whatever she liked, she could not get rid of her sense of loneliness and emptiness. Her marriage no longer gave her happiness. That was why she wanted a divorce, hoping that this would solve her problems.

During joint sessions, it became clear that her husband was a good, responsible man, who really loved and adored his wife. He thought that by working hard, he could provide more financial security and a better future. As a result of meaning therapy, he decided family was more important than money, and that he needed to better manage work-family balance; as a result, he drastically cuts down his projects so that he could spend more time at home. Jackie discovered that her depression was because she was bored with life and did not have an outlet for her love for creative work. She decided to return to college to pursue her interests in internal design. As a result of the above changes, she no longer needs antidepression medication or desires a divorce.

### Case two

Oscar suffers from anxiety. He is a very successful medical professor teaching in an Ivy league university. His main problem is that his 10-year-old son has Type One diabetes, and he feels guilty for his inability to help his son medically in spite of all the honors and awards he has received in his medical field. In addition, he also suffers from his inability to see his son as much as he wants, because his estranged wife (now living in separation) manipulates his visiting time in order to squeeze out more money from him. His previous psychologist advised him to divorce his wife and win the child custody case. But he is reluctant to go to court, because of his concern for his son’s wellbeing. These problems caused him immense pain and anxiety.

Existential answers for Oscar’s problems revolved around the following themes: (a) the Stoic wisdom of changing ourselves rather than changing others (e.g., Aurelius, 2016); (b) cultivating the wisdom of loving his son, but with some emotional detachment so that he would not suffer so intensely; (c) treating his wife with kindness and forgiveness, even though she remains a manipulative and deceitful woman (he wants to believe that love will eventually prevail over evil); and (d) learning to endure the pain with joy and gratitude, because his suffering has brought him closer to God and made him a very successful surgeon because of his extraordinary skills and compassion toward patients. Oscar finds stoic philosophy most helpful in its emphasis on doing what is within his control and what matters most to him. In addition, he finds some inner peace from the principle of acceptance, enduring and praying to God for what is beyond his control.

Space would not allow me to provide more cases. My clients over the last 30 years include successful movie stars, lawyers, physicians, scientists, professors, bankers, CEOs; these individuals possess everything people can only dream of, and yet they still suffer in their private hell, such marital problems, work stress, inner emptiness, and disillusion with life. Therefore, there is the need for a more holistic and meaning-centered narrative for mental health.

## Varieties of suffering in the clinical setting: a new conceptualization

As illustrated by Wong’s examples, many clinical cases are simply normal human reactions to the inescapable sufferings from any one or any combination of the four sources of suffering. We propose that most psychological disorders can be contributed to different types of deficiencies, such as:

1. Deficiency in meeting one’s basic physical needs, such sleep, food, or exercise (Columbia University Department of Psychiatry, 2022).
2. Deficiency in caring for the soul’s yearning or spiritual meaning for hope (for a meaningful future), love (loving relationships with others) and faith in protection and help from God or a higher power (Wong, 2023b).
3. Deficiency in emotional regulations (temper tantrum, frequent mood swing, or lack of emotional intelligence) and self-control and discipline (indulgence in pleasure, addiction, or bad habits such as laziness and gluttony).
4. Deficiency in meaning attribution (exaggerated common attribution biases, such as claiming credit for success and blaming others for failure).
5. Deficiency in responsibility for one’s wellbeing and future in addition to failing to take responsibility for one’s words and deeds (Arslan and Wong, 2022).
6. Deficiency in relational skills, such as listening and speaking truthfully and clearly.
7. Deficiency in basic human decency or virtues, such as honesty, integrity, and kindness.
8. Deficiency in coping resources and skills (Wong et al., 2006).
9. Deficiency in endurance and tolerance of suffering and people one does not like (Wong, 2004).
10. Deficiency in life intelligence (LQ; Wong, 2017) or existential intelligence (Gardner, 2020).

## Integrative meaning therapy

The above examples illustrate that most inorganic psychological difficulties can be re-conceptualized as existential concerns and difficulties in coping with various suffering in life. Therefore, integrative meaning therapy (IMT; Wong, 2010, 2016b, 2020b) seems most appropriate because it focuses on the fundamental human needs for meaning, relationship, and spiritual faith, with the human quest for meaning as its central organizing construct, and inner peace as its desirable outcome. IMT reduces the stigma of mental illness because it focuses on unleashing peoples’ natural power of meaning for healing and flourishing.

Meaning is one of core experiences of human existence. The important role of meaning and purpose for our wellbeing is supported by a mountain of empirical research (e.g., Wong, 2012b; Hicks and Routledge, 2013). At present, many people are wrestling with finding meaning and purpose in their work, marriage, or life in general.

As illustrated by the first case study, one’s primary need for meaning is replaced or suppressed by one’s blind pursuit of happiness and success. Ironically, research has shown that such pursuit is a main source of suffering (Schumaker, 2006; Wong, 2007; Zerwas and Ford, 2021). In addition, toxic positivity has attracted increasing public attention (Scully, 2020; Kaufman, 2021; Princing, 2021; Villines, 2021; Cain, 2022).

The advantages of cultivating a meaning-mindset (Wong, 2011) includes: (1) allowing one to facilitate the discovery of meaning in situations and in one’s life overall; (2) adding a spiritual perspective to everyday activities; (3) allowing an individual to orient themselves to the values of eudemonia and self-transcendence; (4) contributing to personal growth and becoming a fully functioning person; and (5) increasing one’s likelihood of success in having a meaningful purpose.

The good news is that research has shown that meaning is an antidote to the perils of pursuit of happiness and success, when meaning is defined as self-transcendence reorientation (Frankl, 1988; Wong, 2014; Wong et al., 2021a). Self-transcendence can be illustrated by the following widely cited saying from Dalai Lama: “Our prime purpose in this life is to help others. And if you cannot help them, at least do not hurt them.”

IMT focuses on meaning-centered coping which includes (a) finding benefits or lessons from suffering, (b) leaning to accept and transcend inescapable suffering, (c) praying to God or a Higher Power for help, (d) reframing suffering into something more manageable and positive, (e) linking suffering to some meta narrative or mythology, and (f) integrating suffering with something positive or meaningful (Wong et al., 2006; Eisenbeck et al., 2022). Meaning is a common factor in all kinds of therapies (Vos, 2018). Here are 10 characteristics of a meaning-centered psychotherapist:

1. Holds a hopeful view of every client and treats them with respect and dignity.
2. Makes effective use of the self—the therapist is the therapy.
3. Help clients move toward both healing and wellbeing simultaneously.
4. Sees both the big picture and situational problems.
5. Integrates different modalities around the central construct of meaning.
6. Integrates the art and science of meaningful living.
7. Considers meaning as both personally and socially constructed.
8. Empowers clients to take personal responsibility to develop their potentials.
9. Equips clients with skills of making the right decision and effective coping.
10. Takes a holistic view of wellbeing, including spiritual wellbeing.

In sum, IMT involves how to manage the three broad themes of human existence: (1) How to live a fulfilling and meaningful life, (2) How to become better and stronger though overcoming and transforming suffering into something meaningful, and (3) How to love and relate well to others in a multicultural society (Wong, n.d.b).

## Total wellbeing and why the best possible life is a deep life

From the perspective of EPP, we can enjoy total wellbeing when we are able to transcend our limitations, suffering, and cultural differences. In doing so, we can enjoy living a meaningful life involving all four dimensions of our personhood – bio, psycho, social, and spiritual.

According to the American Psychological Association (n.d.), wellbeing is defined as “a state of happiness and contentment, with low levels of distress, overall good physical and mental health and outlook, or good quality of life.” The World Health Organization (2021) has a broader conception of wellbeing beyond physical and mental health as follows:

Well-being is a positive state experienced by individuals and societies. Similar to health, it is a resource for daily life and is determined by social, economic and environmental conditions. Well-being encompasses quality of life and the ability of people and societies to contribute to the world with a sense of meaning and purpose. Focusing on well-being supports the tracking of the equitable distribution of resources, overall thriving and sustainability. A society’s well-being can be determined by the extent to which they are resilient, build capacity for action, and are prepared to transcend challenges (World Health Organization, 2021).

Thus, it calls for total mobilization of all sections and all citizens to be involved in actions of promoting wellbeing in societies, in which all people can enjoy some good quality of life. Toward this goal, we have developed a tripartite meaning management model of focusing on managing three existential universals for sustainable wellbeing: meaningful living, meaningful suffering, and meaningful relationships in a multicultural society.

Research has also shown that illness can be viewed as a spiritual phenomenon according to Dame Cicely Saunders’ ground-breaking concept of total suffering as comprising physical, emotional, social, and spiritual sources of pain (Balboni and Balboni, 2018). By the same token, we can also have total wellbeing, which includes the spiritual-existential source of wellbeing (Wong, 2023b).

**Figure 1:**
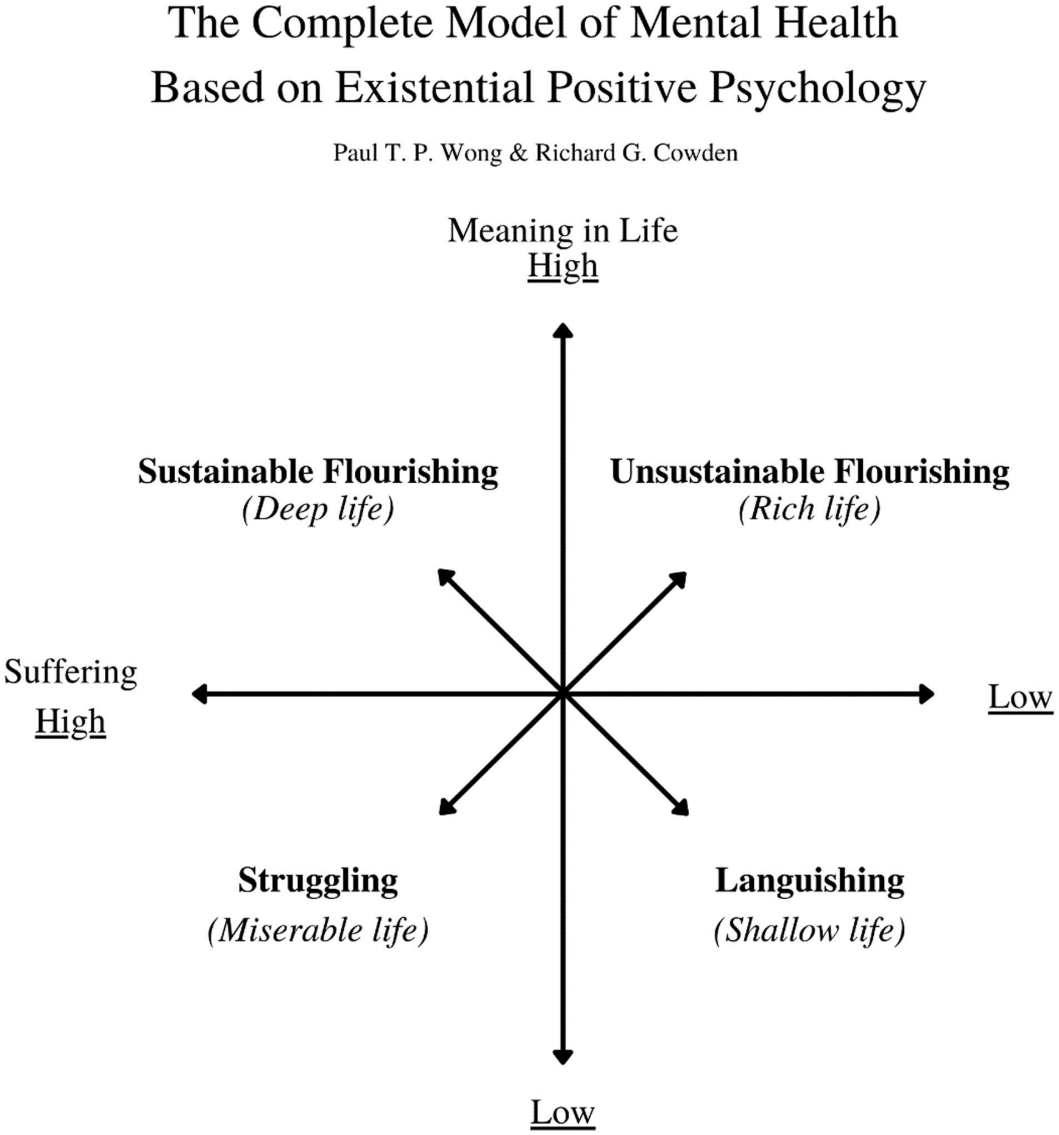
The complete model of mental health based on existential positive psychology.

A complete model of mental health depends on how well we manage suffering, and to what extend we embrace meaning. Figure 1 explains both the importance of suffering and meaning as well as the need for balance.

**Figure 2:**
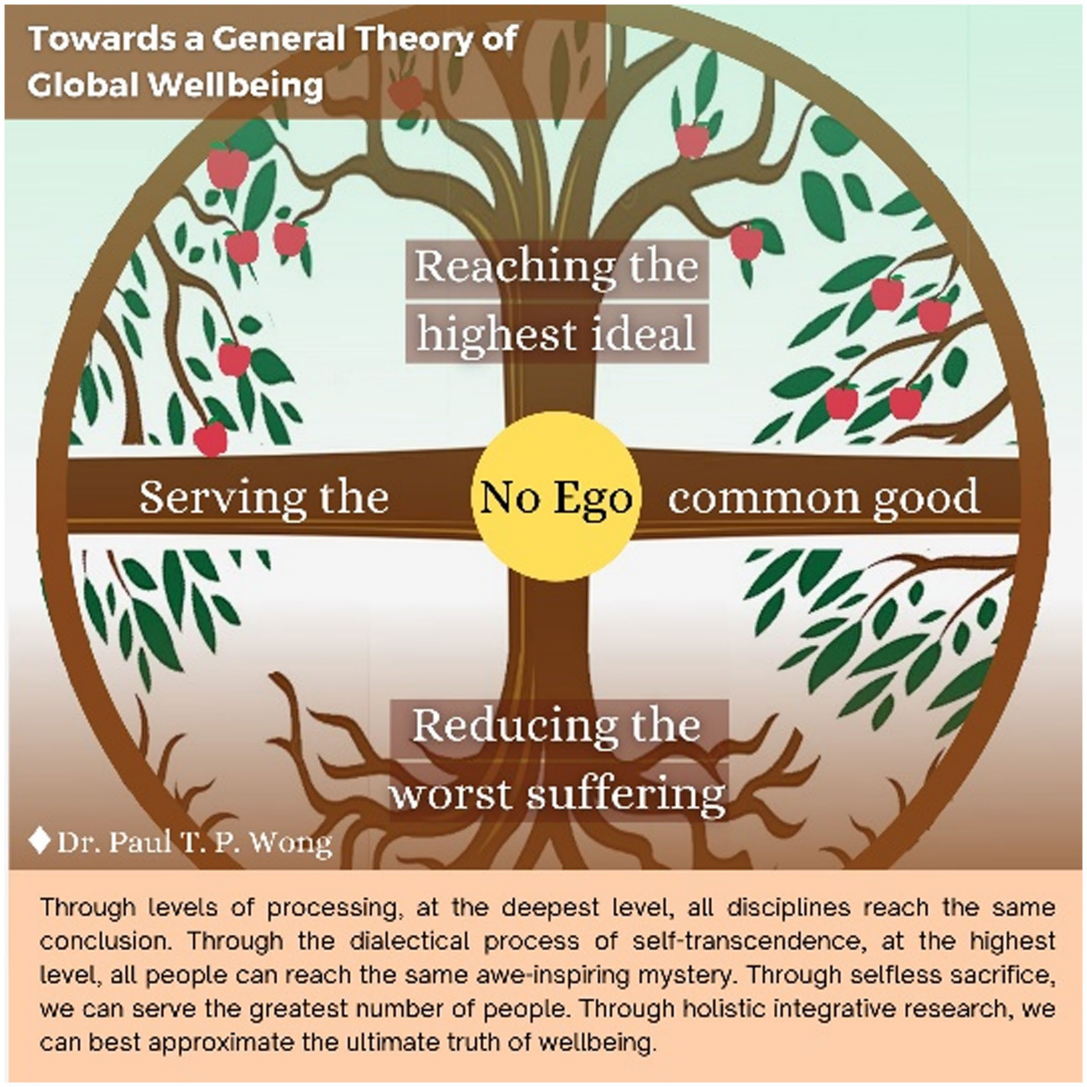
Towards a general theory of global wellbeing.

Regarding the four quadrants, in times like this, when life is full of suffering and stress, the best possible life is a deep life or sustainable flourishing. It may sound counter-intuitive because we instinctively avoid suffering. Figure 2 provides the reasons why suffering is necessary for a deep life.

The second-best life is where the privileged can avoid most of the pain common folks suffer from and engage in all kinds of desirable experiences. This kind of life has been described as the rich life. According to Oishi and Westgate (2022), “Unlike happy or meaningful lives, psychologically rich lives are best characterized by a variety of interesting and perspective-changing experiences.” (p. 790).

But a rich life is unsustainable in the long run, because when one encounters a tough patch of life, struck by unexpected tragedy or trauma, one does not have the solid foundation or resilience to maintain their rich lifestyle.

The quadrant of languishing refers to the ordinary shallow life of the eat-work-sleep cycle. A boring, meaningless existence can be described as a shallow life. “Happiness without meaning characterizes a relatively shallow, self-absorbed or even selfish life, in which things go well, needs and desire are easily satisfied, and difficult or taxing entanglements are avoided” (Baumeister et al., 2013).

The last quadrant refers to the worst possible life, full of suffering and devoid of meaning. It can be called a wasted life or miserable life. A miserable life may be a better description of a living hell without meaning transformation.

Our tripartite model of meaningful living, meaningful suffering, and multicultural relationship can also be translated into the evolutionary psychology of the pain-brain-culture model of wellbeing. From an evolutionary perspective, the main thing animals or human beings have to contend with is danger, pain, or death in order to stay alive. That is why learning how to cope with painful experiences or suffering is a matter of survival and striving, not a matter of pathology or sickness. Meaning and happiness are necessary to make life worth living in order to prevent us from committing suicide or giving up (quiet suicide). This is the logic Soper (2020) and Wong (2022) have argued for.

## Conclusion

The main contribution of this paper is threefold: it explains the need to incorporate suffering as an important factor for sustainable wellbeing, the need for IMT and learning how to live a meaningful life in times of adversity, and the importance of spiritual-existential wellbeing.

We have made the case that suffering is necessary for sustainable wellbeing and flourishing. If we focus only on the negative events in our life, we will be swallowed up by the black hole of depression and anger. However, if we focus on the meaning of suffering and learn to see light or be the light in the darkest hours though practicing hope, love, and faith, we will be strengthened and blessed.

As a new narrative beyond the medical model, our EPP framework encourages the following new trends which may help resolve the current mental health crisis.

1. From a symptom-based approach to a holistic approach to total wellbeing.
2. From clinical treatment to practical guidelines for living fully and vitally.
3. From adhering to a particular school of thought to integrating multiple modalities.
4. From Western-ethnocentric psychology to multicultural and indigenous psychology.
5. From depending only on the medical profession to including educational, and other social institutions.

## Data availability statement

The original contributions presented in the study are included in the article/supplementary material, further inquiries can be directed to the corresponding author.

## Ethics statement

Ethical review and approval was not required for the study on human participants in accordance with the local legislation and institutional requirements. Written informed consent for participation was not required for this study in accordance with the national legislation and the institutional requirements.

## Author contributions

PTPW drafted the manuscript. DL conducted a literature review and reviewed the draft manuscript. All authors contributed to the article and approved the submitted version.

## Conflict of interest

The authors declare that the research was conducted in the absence of any commercial or financial relationships that could be construed as a potential conflict of interest.

## Publisher’s note

All claims expressed in this article are solely those of the authors and do not necessarily represent those of their affiliated organizations, or those of the publisher, the editors and the reviewers. Any product that may be evaluated in this article, or claim that may be made by its manufacturer, is not guaranteed or endorsed by the publisher.

## Footnotes

^1^The case illustrations provided here are fictional characters based on similar cases. Their personal characteristics and identities are disguised so that no one can identify a real person from these case adaptations.
